# Major bleeding and thromboembolic complications associated with antithrombotic treatment in patients with atrial fibrillation/flutter and incident cancer

**DOI:** 10.1016/j.rpth.2025.102697

**Published:** 2025-02-05

**Authors:** Gordon Chu, Nienke van Rein, Menno V Huisman, Lars Pedersen, Henrik T. Sørensen, Suzanne C. Cannegieter, Frederikus A. Klok

**Affiliations:** 1 Department of Medicine–Thrombosis and Haemostasis, Leiden University Medical Centre, Leiden, the Netherlands; 2 Department of Clinical Epidemiology, Aarhus University Hospital and Aarhus University, Aarhus, Denmark; 3 Department of Clinical Epidemiology, Leiden University Medical Centre, Leiden, the Netherlands; 4 Department of Clinical Pharmacy and Toxicology, Leiden University Medical Centre, Leiden, the Netherlands; 5 Center for Population Medicine, Aarhus University Hospital and Aarhus University, Aarhus, Denmark

**Keywords:** anticoagulants, atrial fibrillation, hemorrhage, neoplasms, thromboembolism

## Abstract

**Background:**

Anticoagulant management of patients with atrial fibrillation with active cancer is complex because cancer increases the risk of thrombosis as well as bleeding. Previous studies have investigated the impact of any type of cancer, while outcomes may differ per specific type. We performed the present study to provide more insight into the impact of specific types of cancer on clinical outcomes.

**Objectives:**

We examined major bleeding (MB) and thromboembolism (TE) rates associated with antithrombotic treatment in patients with atrial fibrillation/flutter (AF) who develop cancer and examined whether cancer type affected MB and TE risks.

**Methods:**

This Danish population-based cohort study included all patients aged ≥ 50 years discharged with incident AF between January 1, 1995, and December 31, 2016, and identified those who subsequently developed cancer. Data on cancer type, outcomes, and antithrombotic exposure were obtained from hospital and drug prescription databases. Follow-up continued from the time of cancer diagnosis until the occurrence of an outcome or the end of the 2-year follow-up. Incidence rates (IRs) per 100 patient-years and adjusted hazard ratios with corresponding 95% CIs were calculated using Cox regression.

**Results:**

A total of 22,996 patients with AF with subsequent incident cancer were identified. These patients had higher MB (IR, 5.36 [95% CI, 5.09-5.64] vs 2.27 [95% CI, 2.22-2.32]) and TE (IR, 3.91 [95% CI, 3.68-4.15] vs 2.71 [95% CI, 2.66-2.76]) rates than those without cancer. The higher MB rate was observed across all antithrombotic exposure categories. Urogenital (IR, 6.43 [95% CI, 5.94-6.95]) and intracranial cancer (IR, 6.36 [95% CI, 3.85-9.76]) demonstrated the highest MB rates; hematologic (IR, 4.92 [95% CI, 4.12-5.82]) and gastrointestinal cancer (IR, 4.82 [95% CI, 4.31-5.36]) had the highest TE rates. A particularly high MB rate was observed in patients with AF with gastrointestinal cancer and triple antithrombotic therapy (IR, 39.0 [95% CI, 15.5-79.1]).

**Conclusion:**

Patients with AF with certain incident cancer types experienced higher rates of MB and TE than those without cancer. Dual/triple antithrombotic therapy in patients with AF with incident cancer was associated with high bleeding rates, particularly with gastrointestinal cancer.

## Essentials

• Complications of antithrombotic therapy (ATT) in atrial fibrillation and cancer are understudied.
• This Danish population-based study presents major bleeding and thromboembolism rates of ATT.
• Higher complication rates of ATT were observed in those with incident cancer than those without.
• The degree of additional risk depended on the type of cancer and certain combinations of ATT.

## 1 Introduction

The anticoagulant management of patients with atrial fibrillation with active cancer is complex because cancer increases the risk of arterial and venous thromboembolism (TE), as well as bleeding [1,2]. Factors contributing to this additional risk include the cardiovascular toxicity of anticancer treatment (eg, vascular damage), cancer-associated complications, comorbidities, and hypercoagulability [1,2]. Moreover, patients with certain cancer types are prone to developing thromboembolic and/or bleeding complications [[2], [3], [4], [5], [6]].

Current atrial fibrillation guidelines recommend a risk factor-based approach using the CHA_2_DS_2_-VASc (Congestive HF, Hypertension, Age **≥**75 years, Diabetes mellitus, Stroke/TIA/TE, Vascular disease, Age 65 to 74 years, female Sex Category) stroke risk score to identify patients in whom anticoagulants should be initiated. Direct oral anticoagulants (DOACs) are preferred to vitamin K antagonists (VKAs) [7,8]. Notably, no specific recommendations regarding anticoagulant treatment have been provided for patients with atrial fibrillation with active cancer because the landmark DOAC trials excluded this patient category [[9], [10], [11], [12]]. Moreover, subsequent cohort studies have evaluated primarily patients with atrial fibrillation with any diagnosis of cancer (ie, irrespective of the time interval between the atrial fibrillation and cancer diagnosis) and often have not considered the type of cancer or the antithrombotic management strategy used during follow-up [4,6,[13], [14], [15], [16], [17], [18], [19]]. Therefore, the clinical implications of different treatment strategies are unknown according to antithrombotic treatment type and cancer type due to a lack of data on thromboembolic and major bleeding complications in patients with atrial fibrillation/flutter (AF) who develop cancer.

We, therefore, performed a nationwide cohort study in patients with prevalent atrial fibrillation who developed incident cancer to determine the major bleeding and thromboembolic risks associated with different antithrombotic treatment strategies in different cancer types.

## 2 Methods

### 2.1 Settings and database

Denmark has a free tax-supported health care system [20]. The data sources used in this study were the Danish National Patient Registry, the Danish National Prescription Registry, and the Danish Registry of Causes of Death [[21], [22], [23], [24]]. All Danish residents are included in these national population-based health and administrative registries and are identified by their unique civil personal registration numbers [25].

The Danish National Patient Registry contains data on all inpatient hospitalizations since 1977 and on all hospital specialist outpatient clinic and emergency department visits since 1995, including the dates of visit, admission and discharge, the discharge diagnoses, and selected treatments. Diagnoses are coded according to the International Classification of Diseases (ICD), Eighth Revision (1977-1993), and Tenth Revision (ICD-10; since 1994) [26]. The Danish National Prescription Registry contains information on all prescriptions dispensed at community pharmacies in Denmark since 1995 [27]. The data include the date of dispensing, the quantity of drugs dispensed, and the Anatomical Therapeutic Chemical code of the dispensed drug. Finally, the Danish Registry of Causes of Death contains information on all deaths in Denmark since 1943, which has been computerized since 1970. Data include the dates and the causes of death, classified by ICD-10 codes [23].

### 2.2 Study population

We identified all patients aged 50 years or older with a first-time primary or secondary inpatient or outpatient discharge diagnosis of AF, registered in the Danish National Patient Registry between January 1, 1995, and December 31, 2016. Patients with an AF diagnosis in an acute setting (eg, emergency department) were not eligible for inclusion. A diagnosis of AF has a positive predictive value of 99% in the Danish National Patient Registry [28]. From the AF population, we identified all patients with a subsequent new cancer diagnosis. Follow-up started from the date of a new cancer diagnosis (index date). To ensure that cancer was not diagnosed simultaneously with AF, only patients with cancer diagnosed 30 days after AF diagnosis were included. We stratified cancer by type: gastrointestinal, respiratory and intrathoracic, breast, urogenital, intracranial, hematologic, skin, and other cancers. The ICD codes for AF and cancer are listed in the Supplementary Tables S1 and S2.

To study the association between cancer and the studied outcomes, we created a comparison cohort by matching the patients with AF with active cancer with patients with AF without cancer on the basis of the number of days between the cancer diagnosis and the previous AF diagnosis (±10 days) in a 1 to 10 ratio. Similarly, follow-up started at the matched index date.

### 2.3 Exposure

Data on antithrombotic drug exposure were obtained from the Danish National Prescription Registry according to the Anatomical Therapeutic Chemical classification codes listed in the Supplementary Table S3. The following exposure categories were identified: no antithrombotic treatment; monotherapy with a VKA; monotherapy with a DOAC; monotherapy with an antiplatelet agent; dual therapy with a VKA or DOAC and 1 antiplatelet drug; dual antiplatelet therapy; and triple therapy with a VKA or DOAC and 2 antiplatelet drugs.

Antithrombotic drug exposure was evaluated as a time-dependent variable. Patients were considered to have been exposed starting from the day on which their prescription for an antithrombotic drug was filled. The length of exposure was calculated according to the number of pills collected divided by the dosing regimen. For VKA specifically, the length of exposure was assumed to be 90 days by prescription. Drugs for chronic conditions are rarely provided for >3 months at a time in Denmark. Although low-dose aspirin was available over the counter, patients receiving long-term treatment usually obtain a prescription for reimbursement purposes [29,30]. To account for prescription filling delays and the duration of action of individual drugs, we included a 14-day washout period.

### 2.4 Outcomes and comorbidities

The Danish National Patient Registry and the Danish Registry of Causes of Death were used to collect the following outcomes of interest: major bleeding, ischemic stroke, myocardial infarction, systemic embolism, venous TE, and all-cause mortality. These outcomes were required to have been registered as either a hospital admission with a primary or secondary discharge diagnosis of the outcome of interest or as the primary cause of death.

Comorbidities were defined as diagnoses present at or before the index date. The following comorbidities were of interest: ischemic heart disease, heart failure, valvular heart disease, hypertension, ischemic stroke, systemic embolism, diabetes, liver disease, renal failure, and anemia. Previous studies have demonstrated good validity of the comorbidities and outcomes diagnoses registered in the Danish National Patient Registry [[31], [32], [33], [34], [35]]. The CHA_2_DS_2_-VASc score was calculated according to these diagnostic codes [36]. The ICD, Eighth Revision, and ICD-10 codes of the outcomes and comorbidities of interest are listed in the Supplementary Table S4.

### 2.5 Statistical analysis

Person-time was calculated from the date of the cancer diagnosis (index date) for patients with AF with cancer or from the matched index date for patients with AF without cancer until the occurrence of an outcome of interest, death, or the end of the study period (December 31, 2016), whichever occurred first, with a maximum follow-up of 2 years. In calculating follow-up time until a major bleed or another outcome, we did not consider the occurrence of the other outcomes. For example, if a patient experienced both an ischemic stroke and a major bleeding event, we calculated separate follow-up times for each analysis. Thus, all follow-ups from the diagnosis of cancer to the first major bleeding event were included in the analysis of major bleeding, and all follow-ups until the first ischemic stroke were included in the ischemic stroke analysis. Incidence rates (IRs) per 100 person-years of the outcomes of interest were calculated by exposure group, and subsequent stratification by cancer type was performed.

To compare the thromboembolic and major bleeding rates of the various exposure categories between patients with AF with active cancer and matched patients with AF without active cancer, we estimated hazard ratios (HRs) and 95% CIs with Cox proportional hazards model with time-dependent variables (ie, antithrombotic drug exposure) by including patients with no malignancy and no antithrombotic treatment in the reference group. HRs were adjusted for age, sex, ischemic heart disease, valvular heart disease, diabetes, hypertension, liver disease, and kidney failure. These analyses were repeated, stratified by cancer type, to compare the thromboembolic and major bleeding rates between patients with AF with specific cancer types and no cancer.

## 3 Results

Between January 1, 1995, and December 31, 2016, 22,996 patients with prevalent AF who developed incident cancer were identified: 8033 (35%) were female, and the mean age was 72 years (SD, 9). The mean CHA_2_DS_2_-VASc score was 3.5 (SD, 1.7). The most common comorbidities were hypertension, which was present in 10,453 patients (45%), and ischemic heart disease, which was present in 8919 (39%) patients. A total of 18% and 25% of patients had a prior history of ischemic stroke and major bleeding, respectively. The baseline characteristics are listed in Table 1. The most frequently observed cancer types were urogenital (30%), gastrointestinal (26%), respiratory (19%), breast (9%), hematologic (9%), skin (4%), and primary intracranial (1.6%) cancers. The remaining cancer types contributed only 1.7%.

**Table 1:** Baseline characteristics of all patients in Denmark ≥50 years of age with a first-time primary or secondary hospital inpatient or outpatient discharge diagnosis of atrial fibrillation or flutter between January 1, 1995, and December 31, 2016, and with an active cancer diagnosis. Data are stratified by type of therapy.

Patient characteristics	Matched AF/AFL patients without cancer, *n* (%)	AF/AFL patients with cancer, *n* (%)	No antithrombotic treatment, *n* (%)	Monotherapy			Dual therapy			Triple therapy
				VKA *n* (%)	DOAC, *n* (%)	Antiplatelet, *n* (%)	Dual antiplatelet, *n* (%)	VKA + antiplatelet, *n* (%)	DOAC + antiplatelet, *n* (%)	VKA/DOAC + dual antiplatelet, *n* (%)
Patients, *n*	259,458	22,996	8548 (37)	6316 (27)	1221 (5.3)	4571 (20)	571 (2.5)	1531 (6.7)	136 (0.59)	102 (0.44)
General characteristics										
Mean age (SD), y	70 (11)	72 (9)	71 (10)	71 (8)	72 (9)	74 (10)	75 (9)	71 (8)	73 (8)	72 (9)
Female sex	112,042 (43)	8033 (35)	3179 (37)	1984 (31)	426 (35)	1792 (39)	194 (34)	1132 (74)	38 (28)	21 (21)
CHA_2_DS_2-_VASc	3.3 (1.8)	3.5 (1.7)	3.2 (1.7)	3.4 (1.7)	3.5 (1.7)	3.8 (1.7)	5.0 (1.5)	4.0 (1.6)	3.9 (1.7)	4.7 (1.6)
Type of cancer										
Respiratory and intrathoracic cancer	-	4293 (19)	1580 (18)	1078 (17)	232 (19)	932 (20)	123 (21)	294 (19)	29 (1.9)	25 (25)
Breast cancer	-	2108 (9.2)	786 (9.2)	577 (9.1)	117 (9.6)	467 (10)	46 (8.1)	106 (6.9)	6 (0.39)	3 (2.9)
Urogenital cancer	-	6910 (30)	2443 (29)	2020 (32)	354 (29)	1332 (29)	178 (31)	521 (34)	32 (2.1)	30 (29)
Gastrointestinal cancer	-	6083 (26)	2369 (28)	1631 (26)	322 (26)	1158 (25)	145 (25)	381 (25)	49 (3.2)	28 (27)
Skin cancer	-	902 (3.9)	324 (3.8)	282 (4.5)	49 (4.0)	160 (3.5)	24 (4.2)	52 (3.4)	6 (0.39)	5 (4.9)
Intracranial cancer	-	360 (1.6)	135 (1.6)	96 (1.5)	28 (2.3)	60 (1.3)	10 (1.8)	30 (2.0)	0 (0.00)	1 (0.98)
Hematological cancer	-	2012 (8.7)	786 (9.2)	546 (8.6)	107 (8.8)	393 (8.6)	40 (7.0)	122 (8.0)	12 (0.78)	6 (5.9)
Other malignancies	-	391 (1.7)	152 (1.8)	106 (1.7)	13 (1.1)	78 (1.7)	9 (1.6)	27 (1.8)	2 (0.13)	4 (3.9)
Comorbidities										
IHD	92,440 (36)	8919 (39)	2846 (33)	2024 (32)	354 (29)	2306 (50)	329 (58)	916 (60)	73 (54)	71 (70)
Valvular heart disease	27,707 (11)	2551 (11)	833 (9.7)	809 (13)	100 (8.2)	471 (10)	54 (9.5)	254 (17)	20 (15)	10 (9.8)
Hypertension	108,394 (42)	10,453 (45)	3371 (39)	3003 (48)	697 (57)	2115 (46)	321 (56)	791 (52)	92 (68)	63 (62)
Diabetes mellitus	35,369 (14)	3781 (16)	1202 (14)	1034 (16)	220 (18)	837 (18)	111 (19)	328 (21)	28 (21)	21 (21)
Liver disease	5326 (2.1)	697 (3.0)	316 (3.7)	145 (2.3)	39 (3.2)	143 (3.1)	12 (2.1)	38 (2.5)	3 (2.2)	1 (0.98)
Renal failure	10,221 (3.9)	1231 (5.4)	476 (5.6)	285 (4.5)	61 (5.0)	261 (5.7)	47 (8.2)	90 (5.9)	8 (5.9)	3 (2.9)
*Previous*										
Stroke	46,361 (18)	4103 (18)	1118 (13)	1060 (17)	238 (19)	868 (19)	378 (66)	350 (23)	34 (25)	57 (56)
Myocardial infarction	37,531 (14)	3552 (15)	1072 (13)	628 (9.9)	112 (9.2)	1069 (23)	176 (31)	421 (27)	30 (22)	44 (43)
Major bleeding	47,996 (28)	5824 (25)	2143 (25)	1546 (24)	298 (24)	1136 (25)	161 (28)	461 (30)	45 (33)	34 (33)

AF, atrial fibrillation; AFL, atrial flutter; DOAC, direct oral anticoagulant; IHD, ischemic heart disease; VKA, vitamin K antagonist.

CHA_2_DS_2-_VASc is a mean (SD).

At the time of cancer diagnosis, 8548 (37%) patients did not receive any antithrombotic treatment. Patients were treated predominantly with VKAs (27%). Single antiplatelet treatment and DOAC monotherapy were prescribed in 20% and 5.3% of patients, respectively, and 10% of patients received combination antithrombotic therapy (dual or triple therapy).

### 3.1 Major bleeding risk in patients with AF with incident cancer by type of antithrombotic treatment and cancer type

**Figure 1:**
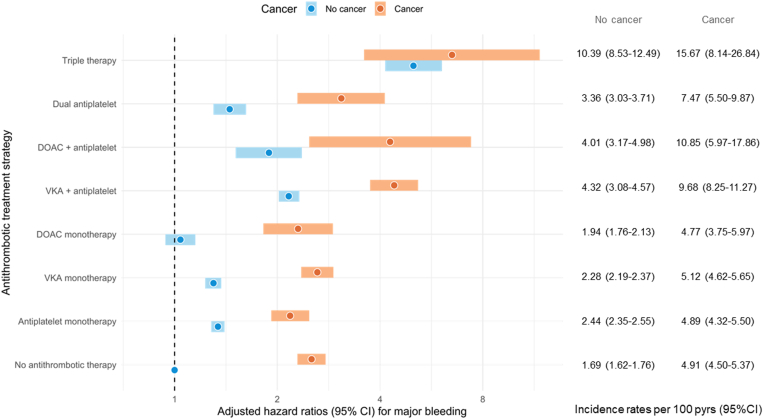
Adjusted hazard ratios for major bleeding by antithrombotic therapy in patients with atrial fibrillation/flutter with and without cancer. Patients with atrial fibrillation/flutter without cancer and without antithrombotic therapy serve as a reference. DOAC, direct oral anticoagulant; VKA, vitamin K antagonist.

The IRs (per 100 patient-years) of major bleeding in patients with and without active cancer were 5.36 (95% CI, 5.09-5.64) and 2.27 (95% CI, 2.22-2.32), respectively, with an adjusted HR (aHR) of 2.11 (95% CI, 1.99-2.23; Table 2 and Supplementary Table S5). The IRs of major bleeding ranged from 4.89 to 39.02 in patients who developed cancer and from 1.69 to 26.31 in patients without cancer, and higher bleeding rates were observed with intensifying combinations of antithrombotic treatment (Supplementary Table S5). The major bleeding event rates in patients with AF with cancer were approximately 2-fold higher than those without cancer across nearly all antithrombotic treatment strategies, including no antithrombotic therapy. Figure 1 illustrates the relative major bleeding risks of the various antithrombotic treatments available in patients with AF with and without cancer.

**Table 2:** Hazard ratios for major bleeding and thromboembolism in patients with atrial fibrillation/atrial flutter and active cancer, stratified by cancer type. Patients with atrial fibrillation/atrial flutter without cancer serve as a reference.

Outcome and cancer type	HR (95% CI)	HR^a^ (95% CI)	HR^b^ (95% CI)
**Major bleeding**			
All cancer vs no cancer	2.30 (2.18-2.43)	2.19 (2.07-2.32)	2.11 (1.99-2.23)
Respiratory cancer	2.73 (2.38-3.13)	2.81 (2.45-3.23)	2.68 (2.34-3.08)
Breast cancer	1.07 (0.85-1.33)	1.24 (0.98-1.57)	1.20 (0.95-1.52)
Urogenital cancer	2.79 (2.55-3.04)	2.44 (2.23-2.67)	2.38 (2.17-2.60)
Gastrointestinal cancer	2.46 (2.22-2.74)	2.28 (2.05-2.53)	2.11 (1.89-2.34)
Skin cancer	1.23 (0.90-1.67)	1.13 (0.83-1.54)	1.08 (0.79-1.47)
Intracranial cancer	2.59 (1.58-4.23)	2.79 (1.70-4.57)	2.81 (1.71-4.62)
Hematological cancer	2.25 (1.87-2.71)	2.18 (1.81-2.62)	2.09 (1.73-2.51)
Other cancer	1.92 (1.29-2.84)	2.03 (1.36-3.01)	1.99 (1.33-2.96)
**TE**			
No cancer vs active cancer	1.42 (1.33-1.51)	1.40 (1.32-1.50)	1.36 (1.27-1.44)
Respiratory cancer	1.68 (1.45-1.96)	1.79 (1.54-2.08)	1.72 (1.48-2.00)
Breast cancer	1.08 (0.89-1.32)	1.03 (0.84-1.27)	1.00 (0.81-1.23)
Urogenital cancer	1.23 (1.10-1.38)	1.22 (1.09-1.37)	1.18 (1.05-1.33)
Gastrointestinal cancer	1.76 (1.57-1.97)	1.69 (1.51-1.90)	1.57 (1.40-1.77)
Skin cancer	0.91 (0.66-1.27)	0.88 (0.63-1.23)	0.87 (0.62-1.21)
Intracranial cancer	1.39 (0.77-2.50)	1.61 (0.89-2.90)	1.64 (0.91-2.96)
Hematological cancer	1.83 (1.52-2.20)	1.83 (1.52-2.21)	1.79 (1.49-2.16)
Other cancer	1.16 (0.74-1.83)	1.25 (0.79-1.97)	1.19 (0.76-1.89)

HR, hazard ratio; TE, thromboembolism.

a Adjusted for age and sex.

b Adjusted for age, sex, ischemic heart disease, valvular heart disease, diabetes, hypertension, liver disease, and kidney failure.

The presence of nearly all cancer types, except for breast and skin cancer, was associated with a 2- to 3-fold increase in major bleeding risk (Table 2). The IRs per 100 patient-years for major bleeding, in descending order, were 6.43 (95% CI, 5.94-6.95) for urogenital cancer, 6.36 (95% CI, 3.85-9.76) for intracranial cancer, 6.26 (95% CI, 5.50-7.09) for respiratory cancer, 5.99 (95% CI, 5.43-6.60) for gastrointestinal cancer, 5.06 (95% CI, 4.25-5.97) for hematologic cancer, 4.70 (95% CI, 3.17-6.67) for other cancer types, 2.73 (95% CI, 2.01-3.60) for skin cancer, and 2.48 (95% CI, 2.00-3.04) for breast cancer (Supplementary Table S5).

Whereas DOAC use was associated with lower major bleeding IRs than VKA use in patients with AF without cancer, higher major bleeding rates were associated with taking DOACs rather than VKAs in patients with AF with urogenital cancer (7.20 per 100 patient-years [95% CI, 5.07-9.86] vs 6.23 [95% CI, 5.36-7.18]), skin cancer (3.51 [95% CI, 0.87-9.11] vs 2.01 [95% CI, 1.04-3.44]), and hematologic cancer (4.58 [95% CI, 1.97-8.86] vs 3.74 [95% CI, 2.48-5.37]; Supplementary Table S5). Furthermore, exceptionally high major bleeding rates were observed in patients with AF treated with triple therapy; the IRs per 100 patient-years for major bleeding during triple therapy were 15.7 (95% CI, 8.14-26.84) and 10.39 (95% CI, 8.53-12.5) in patients with AF with and without cancer, respectively. The highest major bleeding rates were found in patients with AF with gastrointestinal cancer with triple therapy (39.0 per 100 patient-years; 95% CI, 15.5-79.1).

Supplementary Table S6 provides an overview of the sites of bleeding per cancer type and antithrombotic therapy.

### 3.2 Thromboembolic risk in patients with AF with incident cancer by type of antithrombotic treatment and cancer type

**Figure 2:**
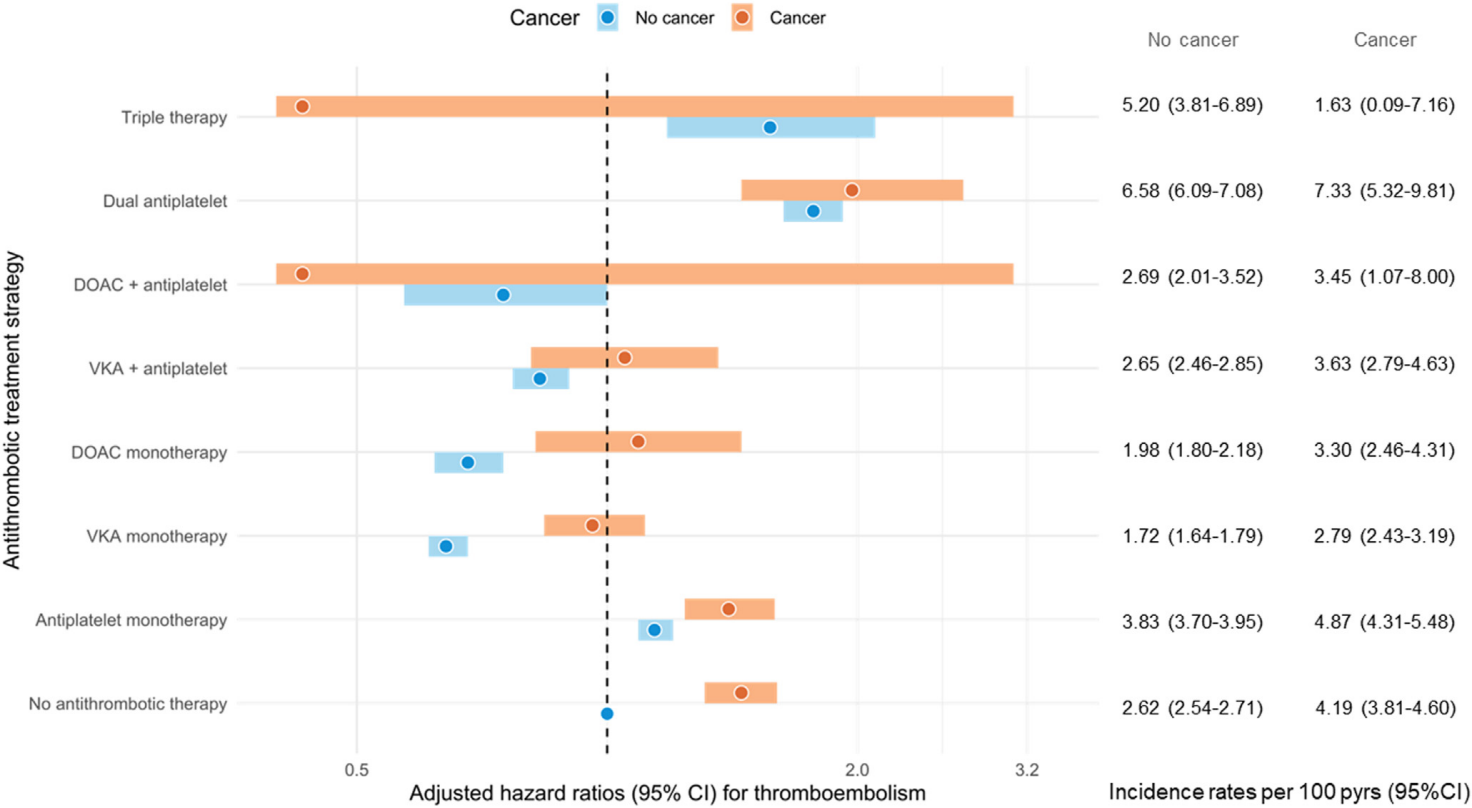
Adjusted hazard ratios for thromboembolism by antithrombotic therapy in patients with atrial fibrillation/flutter with and without cancer. Patients with atrial fibrillation/flutter without cancer and without antithrombotic therapy serve as a reference. DOAC, direct oral anticoagulant; VKA, vitamin K antagonist.

The IRs per 100 patient-years of TE in patients with and without cancer were 3.91 (95% CI, 3.68-4.15) and 2.71 (95% CI, 2.66-2.76), respectively, with an aHR of 1.36 (95% CI, 1.27-1.44; Table 2 and Supplementary Table S7). Patients with active cancer had higher rates of TE than patients without cancer across nearly all treatment strategies; the lowest IRs per 100 patient-years were found for patients taking VKA monotherapy (2.79; 95% CI, 2.43-3.19) and DOAC monotherapy (3.30; 95% CI, 2.46-4.31). These rates were 1.72 (95% CI, 1.64-1.79) and 1.98 (95% CI, 1.80-2.18) for VKA and DOAC monotherapy in patients with AF without cancer, respectively (Supplementary Table S7). Untreated patients with AF with cancer had a nearly 2-fold higher IR for TEs than untreated patients with AF without active cancer (4.19 [95% CI, 3.81-4.60] vs 2.62 [95% CI, 2.54-2.71]), with an aHR of 1.45 (95% CI, 1.31-1.60; Figure 2 and Supplementary Table S7). Figure 2 further illustrates the relative thromboembolic risks by antithrombotic treatment strategy in patients with AF with and without cancer.

The cancer types associated with high thromboembolic risk were hematological cancer (IR, 4.92; 95% CI, 4.12-5.82), gastrointestinal cancer (IR, 4.82; 95% CI, 4.31-5.36), respiratory tract cancer (IR, 4.75; 95% CI, 4.11-5.45), and urogenital cancer (IR, 3.35; 95% CI, 3.00-3.72; Supplementary Table S7). These findings were also reflected in the aHRs for patients with vs without these cancer types, which were 1.79 (95% CI, 1.49-2.16) for hematological cancer, 1.57 (95% CI, 1.40-1.77) for gastrointestinal cancers, 1.72 (95% CI, 1.48-2.00) for respiratory tract cancer, and 1.18 (95% CI, 1.05-1.33) for urogenital cancer (Table 2). Notably, these cancer types were also associated with elevated major bleeding risk.

## 4 Discussion

From this large nationwide cohort study, we obtained data on major bleeding and thromboembolic risk in patients with AF with or without an incident active cancer diagnosis, stratified by cancer type and antithrombotic management strategy. A subsequent cancer diagnosis in patients with prevalent AF was associated with an increased risk of major bleeding and TE, and the extent of the increased risk was associated with cancer type. Specifically, respiratory, urogenital, gastrointestinal, and hematological cancer were associated with elevated risks of major bleeding and TE.

### 4.1 Major bleeding in patients with AF with active cancer

We observed a 2-fold increase in the major bleeding rate associated with the presence of nearly all assessed cancer types. In line with findings from prior studies, the highest major bleeding rates were observed for intracranial, respiratory, and urogenital cancer, whereas no increased bleeding was found for breast and skin cancer [2,4,6,15,16]. Although antithrombotic treatment clearly exacerbated the likelihood of bleeding, the observed 2.5-fold higher major bleeding rate in untreated patients with AF with cancer compared with those without cancer illustrated the tendency toward bleeding due to the active cancer itself. We hypothesize that the invasive anticancer treatments and cancer-associated comorbidities (eg, thrombocytopenia or secondary infections) contributed to the greater bleeding tendency observed in patients with AF with active cancer than in those without cancer [1].

Current guidelines do not provide any strong recommendations regarding the preferred anticoagulant for patients with AF with cancer [7,8,37]. Post hoc analyses of the DOAC trials and observational studies have demonstrated similar or even superior safety profiles for DOACs vs VKAs [13,[16], [17], [18], [19]]. However, conflicting results have been reported regarding possible increased bleeding due to DOACs in gastrointestinal cancers [[38], [39], [40], [41]]. In our observational study, no major differences in major bleeding rates were observed between VKAs or DOACs, regardless of cancer type.

Very high major bleeding rates were observed in patients with AF with active cancer treated with double antiplatelet, double antithrombotic, or triple therapy, and the highest major bleeding IR was found in patients with AF with gastrointestinal cancer receiving triple therapy. This finding prompts the question of whether triple therapy after percutaneous coronary intervention in patients with AF with cancer should be considered. Notably, the data analyzed in this study were obtained in a period in which triple therapy was still recommended in the guidelines for at least 1 to 6 months [42,43]. According to current guidelines, active malignancy within 12 months is considered a major bleeding risk, and triple therapy is recommended for as long as 1 week or until discharge; the duration of double antithrombotic therapy is generally shorter, at 6 months instead of 12 months [7,44,45]. Nonetheless, the results of this study warrant a thorough consideration of the necessity for and duration of double antithrombotic and triple therapy in patients with AF with active cancer.

### 4.2 Thromboembolic risks in patients with AF with active cancer

Nearly all cancer types were associated with an elevated TE rate in patients with AF who developed cancer. The highest thromboembolic rates were observed in patients with hematological, respiratory, intracranial, or gastrointestinal cancer, which is in agreement with findings from previous studies reporting on the increased thrombogenicity in different cancer types [3,5]. Prior studies in patients with AF with cancer have not observed as strong an association and have reported only an elevated thromboembolic risk associated with select cancer types, such as in respiratory, uterine, or pancreatic cancer, or in patients with a recent (<1 year prior) cancer diagnosis [2,4,17,46]. These studies probably did not detect an association because they included patients with AF with prior cancer. The highest thromboembolic risks were observed in patients with AF or VTE and recent (ie, <1 year prior) or active cancer rather than in patients with a prior history of cancer, thus reflecting the temporal association between thrombogenicity and cancer activity [4,47].

A comparable or even superior efficacy profile of DOACs compared with VKAs in patients with AF with cancer has been suggested in post hoc and observational studies [19]. In general, we observed comparable thromboembolic IRs between DOACs and VKAs. Of note, a substantial proportion of patients were treated with antiplatelet monotherapy. The combined high rates of major bleeding and TE observed in patients receiving antiplatelet monotherapy warrant caution in prescribing antiplatelet monotherapy in patients with AF with active cancer as a replacement for anticoagulant treatment in an attempt to mitigate bleeding risk.

The CHA_2_DS_2_-VASc score, which has been recommended in contemporary AF guidelines to guide the initiation of anticoagulants in AF, does not include active cancer as a risk factor. Currently, a CHA_2_DS_2_-VASc score of 1 warrants consideration for initiating anticoagulants in patients with AF, and observational studies have reported IRs of TE in untreated patients with AF varying between 0.6 and 1.7 per 100 patient-years [7,[48], [49], [50], [51], [52]]. The IRs for TE observed in untreated patients with AF with cancer were 3-fold higher than those in untreated patients with AF without cancer. However, this finding should be interpreted with caution because we did not account for the CHA_2_DS_2_-VASc score. Patients who were not treated because of a high bleeding risk could similarly have had a high thromboembolic risk because bleeding and thrombotic risk factors largely overlap. Therefore, whether active cancer should be incorporated into the formal assessment for starting anticoagulant treatment in patients with AF throughout the course of active cancer is unclear. Future randomized studies should assess thromboembolic risk in untreated patients with AF with a CHA_2_DS_2_-VASc score of 0 to 1.

### 4.3 Study strengths and limitations

An important feature of this study is the inclusion of AF patients with incident cancer. Because thromboembolic and bleeding risks are associated with cancer activity, estimates of the additional thromboembolic and bleeding risks conferred by cancer are likely to have been underestimated in previous studies, including patients with prior cancer [4,6,13,14]. Moreover, the few studies that have included patients with incident cancer or have limited the time interval between both cancer and AF diagnosis to 6 to 12 months have often excluded patients without any antithrombotic treatment or have not stratified by cancer type [[53], [54], [55]]. In our study, we aimed to address these aforementioned issues by presenting thromboembolic and major bleeding IRs of the various antithrombotic exposure possibilities (including no antithrombotic or combination therapy) while considering the various cancer types. Moreover, our study included substantial numbers of DOAC users and provided data on matched patients with AF without cancer. Finally, because all discharge diagnoses and outpatient pharmacy dispensing are registered in the Danish National Patient Registry and Danish National Prescription Registry, respectively, we were able to obtain large numbers of outcome events and perform subgroup analyses and time-dependent analyses of antithrombotic exposure [21,27,56].

### 4.4 Limitations

Several limitations of this study should be noted. Data on AF type, burden, recurrences, and ablation were or could not be collected. Data on cancer treatment, metastatic status, or remission were or could not be retrieved from the Danish National Patient Registry; the defined follow-up duration of 2 years in which we considered cancer to be active could, therefore, have been overestimated. However, such an overestimation would have diluted the risk estimates determined herein. Furthermore, despite adjustment for several confounders, the differences between the groups cannot be interpreted causally to assess the safety and efficacy of the optimal anticoagulant treatment because several considerations associated with bleeding and thromboembolic risk played roles in the allocation of the treatments (ie, residual confounding by indication). In addition, the multiple stratifications and the resulting small subgroups limited the statistical power of our results to explore or address all possible associations. Moreover, data regarding race, ethnicity, and sociocultural characteristics of the participants were not obtained; this is a limitation to the understanding of the impact of the sociocultural background of the studied population on anticoagulant management in patients with AF and cancer. Future studies should consider these characteristics. Finally, the dispensing data do not provide information on the in-hospital use of low-molecular-weight heparin, the adherence, or lack thereof, to antithrombotic treatment, and temporary (justified) interruptions of antithrombotic treatment during follow-up.

## 5 Conclusion

This study showed that patients with AF and active cancer experienced higher rates of major bleeding and thromboembolic complications than patients with AF without cancer. The highest major bleeding rates were observed for intracranial and respiratory cancer, whereas hematological and respiratory cancer were associated with the highest thromboembolic rates. Very high major bleeding rates were observed in patients with AF with active cancer treated with double antiplatelet, double antithrombotic, or triple therapy.
