# Supplementary material for: Major bleeding and thromboembolic complications associated with antithrombotic treatment in patients with atrial fibrillation/flutter and incident cancer
Source: Res Pract Thromb Haemost. 2025 Feb 5;9(2):102697. doi: 10.1016/j.rpth.2025.102697 (PMC11946762; doi:10.1016/j.rpth.2025.102697)
Supplement: Supplementary material [file mmc1.docx]

**ONLINE SUPPLEMENTARY MATERIAL**

**Supplementary Table S1** ICD-10 codes for atrial fibrillation/flutter and the outcomes of interest

| Outcome of interest | Location/description | ICD-10 codes |
| --- | --- | --- |
| Atrial fibrillation / flutter |  | I48 |
| Bleeding | Heart | I31.2 |
|  | Urine | N02, R31 |
|  | Airways: nose/throat/bronchi | R04 |
|  | Ear |  |
|  | Eye | H11.3, H31.302, H31.303, H31.309, H356, H431 |
|  | Gastrointestinal | K25.0, K25.2, K25.4, K25.6, K26.0, K26.2, K26.4, K26.6, K27.0, K27.2, K27.4, K27.6, K28.0, K28.2, K28.4, K28.6, K29.71, K62.5, K661, K92.0, K92.1, K92.2, I85.01, I85.11, K55.21, K63.81 |
|  | Hemorrhagic stroke | I60, I61, I62 |
|  | Dural bleeds | S064, S065, S066 |
|  | Joints | M250 |
|  | Thorax | J94.2 |
|  | Anemia due to bleeding | D50.0, D62 |
| Arterial thromboembolism (ATE) | Ischemic stroke | I63 - I66 |
|  | Arterial embolism and thrombosis | I74  I74.0 Embolism and thrombosis of abdominal aorta  I74.1 Embolism and thrombosis of other and unspecified parts of aorta  I74.2 Embolism and thrombosis of arteries of upper extremities  I74.3 Embolism and thrombosis of arteries of lower extremities  I74.4 Embolism and thrombosis of arteries of extremities, unspecified  I74.5 Embolism and thrombosis of iliac artery  I74.8 Embolism and thrombosis of other arteries  I74.9 Embolism and thrombosis of unspecified artery |
|  | Myocardial infarction | I21 (I22, I23, I24) |
|  | Renal embolism | N28.0 |
|  | Retinal | H34 |
|  | Spinal cord | G95.11 |
| Venous thromboembolism (VTE) | Pulmonary embolism | I26 |
|  | Phlebitis and thrombophlebitis of femoral vein | I80.1 |
|  | Phlebitis and thrombophlebitis of other unspecified deep vessels of lower extremities | I80.2 |
|  | Acute embolism and thrombosis of axillary vein | I82.A1 |
|  | Acute embolism and thrombosis of deep veins of upper extremity | I82.62 |
|  | Acute embolism and thrombosis of deep veins of lower extremity | I82.4 |
|  | Vena cava | I82.2 |
|  | Vena renalis | I82.3 |
|  | Vena porta | I81 |
|  | Deep vein thrombosis | I82.4, I82.6 |

**Supplementary Table S2.** ICD-10 codes for cancer and their classification in groups of interest

| Group of interest | Cancer type | ICD-10 codes | ICD-8 codes |
| --- | --- | --- | --- |
|  |  |  | Site code |
| Respiratory and intrathoracic cancers | Head and neck: lip, oral cavity and pharynx | C00-C14 | Malignant neoplasm of buccal cavity and pharynx 140–149 |
|  | Nasal, larynx, trachea | C30-C33 |  |
|  | Respiratory | C34 | Malignant neoplasm of respiratory system 160–163 |
|  | Heart | C38 | Heart: N/A |
|  | Other and ill-defined intrathoracic organs | C39 | Malignant neoplasm of other and unspecified respiratory organs  163 |
|  | Mesothelioma | C44 |  |
| Breast cancer | Breast | C50 | Malignant neoplasm of breast 174 |
| Urogenital cancers | Female: reproduction | C51, C52, C53, C54, C55, C56, C57, C58 | Malignant neoplasm of genito-urinary organs 180–189 |
|  | Male: reproduction | C60-C63 |  |
|  | Kidney and urinary | C64, C65, C66, C67, C68 |  |
| Gastrointestinal cancers | Upper GI | C15, C16, C17 | Malignant neoplasm of digestive organs and peritoneum 150–159 |
|  | Lower GI | C18, C19, C20, C21 |  |
|  | Liver, gallbladder, pancreas | C22, C23, C24, C25 |  |
|  | Other and ill-defined digestive organs | C26 |  |
| Skin cancer | Melanoma | C43 | Malignant melanoma of skin 172 |
| Intracranial cancers | Central nervous system and ophthalmic malignancies | C69, C70, C71, C72 | Malignant neoplasm of eye 190  Malignant neoplasm of brain 191  Malignant neoplasm of other parts of nervous system 192 |
| Hematological cancers | Non-solid | C81, C82, C83, C84, C85, C86, C87, C88, C89, C90, C91, C91, C92, C93, C94, C95, C96 | Neoplasms of lymphatic and hematopoietic tissue 200–207  *Omitted 208 and 209 (polycythemia vera and myelofibrosis), because they were not in the C category in ICD-10* |
| Other malignancies | Bone and articular cartilage | C40-C41 | Malignant neoplasm of bone 170  Malignant neoplasm of connective and other soft-tissue 171 |
|  | Thyroid/adrenal/endocrine, neuroendo | C73–75, C7A | Malignant neoplasm of thyroid gland 193  Malignant neoplasm of other endocrine glands 194 |
|  | Thymus | C37 |  |
|  | Kaposi sarcoma | C46 |  |
|  | Peripheral nerves and autonomic nervous system | C47 |  |
|  | Retroperitoneum and peritoneum | C48 |  |
|  | Other connective and soft tissue | C49 |  |
| Excluded | Other and unspecified malignant neoplasm of skin | C44 |  |
|  | Malignant neoplasm without specification of site | C80 |  |
|  | Malignant neoplasm of independent primary multiple sites | C97.9 |  |

**Supplementary Table S3** ATC codes for antithrombotic drugs of interest

| Type | ATC code |
| --- | --- |
| VKA | B01AA03, B01AA04 |
| Dabigatran | B01AE07 |
| Rivaroxaban | B01AE07 |
| Apixaban | B01AF02 |
| Edoxaban | B01AF03 |
| ASA | B01AC06, N02BA1 |
| Clopidogrel | B01AC04 |
| Ticagrelor | B01AC22 |
| Prasugrel | B01AC24 |

Abbreviations: ASA (acetylsalicylic acid), ATC (anatomical therapeutic chemical), VKA (vitamin K antagonists)

**Supplementary Table S4** ICD-10 codes for comorbidities of interest

| Comorbidity | ICD-10 code |
| --- | --- |
| Liver disease | B15-B19, C22,K70-K77, R16.0, R16.2 |
| Renal failure | N17-N19, R34 |
| Ischemic heart disease | I20-I25 |
| Heart failure | I50 |
| Valvular heart disease | I34-I37 |
| Hypertension | I10-I15 |
| Ischemic stroke | I63-I66, 169.3, 169.4 |
| Systemic embolism | I26, I74 |
| Diabetes | E10-E14 |
| Anemia | D63, D64 |

Abbreviation: ICD (international classification of diseas

**Supplementary Table S5** Incidence rates and hazard ratios for major bleeding associated with antithrombotic therapy in patients with AF/AFL **with** **active cancer.** Matched patients with AF/AFL without cancer and without antithrombotic therapy serve as a reference for all HRs.

|  | **No cancer** | | | **Active cancer** | | | **No cancer** | | | **Active cancer** | |
| --- | --- | --- | --- | --- | --- | --- | --- | --- | --- | --- | --- |
|  | MB (n) | Person  years | Incidence rate per 100 pyrs  (95% CI) | MB | Person  years | Incidence rate  (95% CI) | Crude hazard ratio  (95% CI) | Adjusted hazard ratio  (95% CI)* | Crude hazard ratio  (95% CI) | | Adjusted hazard ratio  (95% CI)* |
| **All cancer** | | | | | | | | | | | |
| All patients | 9629 | 423898 | 2.27 (2.22–2.32) | 1447 | 27002 | 5.36 (5.09–5.64) |  |  |  | |  |
| No antithrombotic therapy | 2580 | 152741 | 1.69 (1.62–1.76) | 500 | 10177 | 4.91 (4.50–5.37) | Reference | Reference | 2.83 (2.57–3.11) | | 2.52 (2.29–2.77) |
| Antithrombotic therapy | 7049 | 271158 | 2.60 (2.54–2.66) | 947 | 16825 | 5.63 (5.28–5.99) | 1.53 (1.47–1.60) | 1.34 (1.28–1.40) | 3.25 (3.02–3.50) | | 2.64 (2.46–2.85) |
| Antiplatelet | 2264 | 92620 | 2.44 (2.35–2.55) | 265 | 5421 | 4.89 (4.32–5.50) | 1.44 (1.36–1.52) | 1.18 (1.11–1.25) | 2.82 (2.48–3.20) | | 2.18 (1.92–2.48) |
| VKA | 2644 | 115999 | 2.28 (2.19–2.37) | 383 | 7482 | 5.12 (4.62–5.65) | 1.35 (1.28–1.42) | 1.30 (1.23–1.37) | 2.96 (2.66–3.30) | | 2.62 (2.35–2.92) |
| DOAC | 414 | 21389 | 1.94 (1.76–2.13) | 71 | 1488 | 4.77 (3.75–5.97) | 1.15 (1.04–1.28) | 1.04 (0.94–1.15) | 2.75 (2.17–3.48) | | 2.30 (1.82–2.91) |
| Dual antiplatelet | 375 | 11158 | 3.36 (3.03–3.71) | 45 | 602 | 7.47 (5.50–9.87) | 1.98 (1.78–2.20) | 1.45 (1.30–1.62) | 4.29 (3.19–5.75) | | 3.08 (2.29–4.13) |
| VKA + antiplatelet | 1170 | 27076 | 4.32 (3.08–4.57) | 159 | 1642 | 9.68 (8.25–11.27) | 2.54 (2.37–2.72) | 2.16 (2.02–2.32) | 5.57 (4.75–6.54) | | 4.40 (3.74–5.17) |
| DOAC + antiplatelet | 76 | 1896 | 4.01 (3.17–4.98) | 13 | 120 | 10.85 (5.97–17.86) | 2.37 (1.89–2.96) | 1.89 (1.51–2.36) | 6.17 (3.58–10.64) | | 4.28 (2.48–7.39) |
| VKA/DOAC triple therapy | 106 | 1020 | 10.39 (8.53–12.49) | 11 | 70 | 15.67 (8.14–26.84) | 6.12 (5.06–7.41) | 5.01 (4.14–6.07) | 8.83 (4.89–15.97) | | 6.50 (3.59–11.75) |
| **Respiratory and intrathoracic cancers** | | | | | | | | | | | |
| All patients | 1602 | 72340 | 2.21 (2.11–2.32) | 239 | 3817 | 6.26 (5.50–7.09) |  |  |  | |  |
| No antithrombotic therapy | 426 | 26222 | 1.62 (1.48–1.78) | 83 | 1520 | 5.46 (4.37–6.72) | Reference | Reference | 3.24 (2.56–4.10) | | 3.13 (2.47–3.96) |
| Antithrombotic therapy | 1176 | 46118 | 2.55 (2.41–2.70) | 156 | 2296 | 6.79 (5.78–7.92) | 1.57 (1.40–1.75) | 1.37 (1.23–1.54) | 4.04 (3.36–4.86) | | 3.53 (2.94–4.26) |
| Antiplatelet | 384 | 15932 | 2.41 (2.18–2.66) | 41 | 809 | 5.07 (3.67–6.78) | 1.48 (1.29–1.70) | 1.21 (1.05–1.39) | 3.01 (2.18–4.14) | | 2.54 (1.84–3.50) |
| VKA | 430 | 19317 | 2.23 (2.02–2.44) | 62 | 911 | 6.80 (5.25–8.64) | 1.37 (1.20–1.57) | 1.34 (1.17–1.53) | 4.07 (3.11–5.31) | | 3.88 (2.97–2.07) |
| DOAC | 71 | 3815 | 1.86 (1.46–2.33) | 16 | 244 | 6.57 (3.85–10.33) | 1.14 (0.88–1.46) | 1.04 (0.81–1.34) | 3.94 (2.39–6.49) | | 3.43 (2.08–5.66) |
| Dual antiplatelet | 57 | 1891 | 3.02 (2.30–3.87) | 10 | 92 | 10.82 (5.42–18.98) | 1.85 (1.40–2.44) | 1.37 (1.04–1.82) | 6.33 (3.38–11.86) | | 5.19 (2.77–9.72) |
| VKA/DOAC + antiplatelet | 213 | 4992 | 4.27 (3.72–4.87) | 26 | 231 | 11.26 (7.47–16.16) | 2.62 (2.22–3.09) | 2.25 (1.90–2.66) | 6.65 (4.47–9.88) | | 5.79 (3.88–8.62) |
| VKA/DOAC triple therapy | 21 | 173 | 12.17 (7.68–18.14) | # | # | 10.68 (0.61–47.00) | 7.37 (4.75–11.42) | 5.83 (3.75–9.05) | 6.15 (0.86–43.78) | | 5.29 (0.74–37.65) |
| **Breast cancer** | | | | | | | | | | | |
| All patients | 774 | 33268 | 2.33 (2.17–2.49) | 87 | 3502 | 2.48 (2.00–3.04) |  |  |  | |  |
| No antithrombotic therapy | 222 | 12055 | 1.84 (1.61–2.09) | 24 | 1350 | 1.78 (1.16–2.59) | Reference | Reference | 0.96 (0.63–1.47) | | 1.08 (0.70–1.65) |
| Antithrombotic therapy | 552 | 21213 | 2.60 (2.39–2.83) | 63 | 2151 | 2.93 (2.26–3.71) | 1.41 (1.21–1.65) | 1.27 (1.08–1.48) | 1.59 (1.20–2.10) | | 1.59 (1.19–2.13) |
| Antiplatelet | 174 | 7286 | 2.39 (2.05–2.76) | 19 | 722 | 2.63 (1.62–4.00) | 1.30 (1.06–1.58) | 1.12 (0.91–1.37) | 1.42 (0.89–2.28) | | 1.31 (0.81–2.11) |
| VKA | 210 | 9056 | 2.32 (2.02–2.65) | 21 | 961 | 2.18 (1.38–3.26) | 1.26 (1.04–1.52) | 1.21 (1.00–1.46) | 1.19 (0.75–1.86) | | 1.26 (0.80–2.00) |
| DOAC | 36 | 1624 | 2.22 (1.57–3.02) | 4 | 191 | 2.10 (0.65–4.88) | 1.20 (0.84–1.71) | 1.08 (0.76–1.55) | 1.14 (0.42–3.07) | | 1.16 (0.43–3.14) |
| Dual antiplatelet | 31 | 883 | 3.51 (2.42–4.90) | # | # | 3.98 (0.99–10.32) | 1.90 (1.31–2.77) | 1.51 (1.03–2.21) | 2.16 (0.69–6.74) | | 1.83 (0.58–5.73) |
| VKA/DOAC + antiplatelet | 90 | 2273 | 3.96 (3.20–4.83) | 16 | 198 | 8.10 (4.75–12.74) | 2.15 (1.68–2.75) | 1.88 (1.46–2.42) | 4.40 (2.64–7.30) | | 4.34 (2.59–7.28) |
| VKA/DOAC triple therapy | 11 | 90 | 12.21 (3.34–20.90) | # | # | NP | 6.61 (3.61–12.11) | 6.06 (3.30–11.13) | NP | | NP |
| **Urogenital cancers** | | | | | | | | | | | |
| All patients | 2506 | 110401 | 2.27 (2.18–2.36) | 624 | 9708 | 6.43 (5.94–6.95) |  |  |  | |  |
| No antithrombotic therapy | 688 | 39820 | 1.73 (1.60–1.86) | 205 | 3387 | 6.05 (5.26–6.92) | Reference | Reference | 3.44 (2.94–4.02) | | 2.84 (2.43–3.33) |
| Antithrombotic therapy | 1818 | 70581 | 2.58 (2.46–2.70) | 419 | 4321 | 6.63 (6.01–7.28) | 1.49 (1.36–1.63) | 1.30 (1.19–1.42) | 3.78 (3.35–4.27) | | 2.88 (2.55–3.27) |
| Antiplatelet | 587 | 24261 | 2.42 (2.23–2.62) | 97 | 1941 | 5.00 (4.07–6.06) | 1.40 (1.25–1.56) | 1.15 (1.03–1.28) | 2.85 (2.30–3.52) | | 2.05 (1.65–2.54) |
| VKA | 715 | 30055 | 2.38 (2.21–2.56) | 179 | 2875 | 6.23 (5.36–7.18) | 1.38 (1.24–1.53) | 1.31 (1.18–1.46) | 3.56 (3.02–4.18) | | 2.93 (2.48–3.47) |
| DOAC | 102 | 5547 | 1.84 (1.50–2.22) | 35 | 486 | 7.20 (5.07–9.86) | 1.05 (0.86–1.30) | 0.96 (0.78–1.18) | 4.08 (2.91–5.73) | | 3.35 (2.38–4.71) |
| Dual antiplatelet | 108 | 2867 | 3.77 (3.10–4.52) | 21 | 233 | 9.00 (5.68–13.41) | 2.17 (1.77–2.66) | 1.61 (1.32–1.98) | .511 (3.31–1.89) | | 3.37 (2.18–5.21) |
| VKA/DOAC + antiplatelet | 282 | 7592 | 3.72 (3.30–4.17) | 83 | 753 | 11.02 (8.82–13.57) | 2.15 (1.87–2.47) | 1.83 (1.59–2.10) | 6.29 (5.01–7.90) | | 4.63 (3.68–5.83) |
| VKA/DOAC triple therapy | 24 | 271 | 8.87 (5.78–12.90) | 4 | 32 | 12.44 (3.86–28.90) | 5.00 (3.33–7.51) | 4.10 (2.73–6.17) | 7.07 (2.65–18.89) | | 5.20 (1.94–13.91) |
| **Gastrointestinal cancers** | | | | | | | | | | | |
| All patients | 2355 | 98834 | 2.38 (2.29–2.48) | 403 | 6725 | 5.99 (5.43–6.60) |  |  |  | |  |
| No antithrombotic therapy | 617 | 35634 | 1.73 (1.60–1.87) | 150 | 2685 | 5.59 (4.74–6.53) | Reference | Reference | 3.15 (2.64–3.77) | | 2.59 (2.16–3.09) |
| Antithrombotic therapy | 1738 | 63200 | 2.75 (2.62–2.88) | 253 | 4040 | 6.26 (5.52–7.07) | 1.59 (1.45–1.74) | 1.39 (1.26–1.52) | 3.55 (3.06–4.11) | | 2.72 (2.35–3.15) |
| Antiplatelet | 562 | 21436 | 2.62 (2.41–2.84) | 87 | 1320 | 6.59 (5.30–8.07) | 1.51 (1.35–1.70) | 1.24 (1.11–1.40) | 3.74 (2.99–4.68) | | 2.76 (2.20–3.46) |
| VKA | 646 | 27182 | 2.38 (2.20–2.56) | 99 | 1767 | 5.60 (4.57–6.78) | 1.37 (1.23–1.53) | 1.32 (1.18–1.48) | 3.18 (2.57–3.93) | | 2.63 (2.12–3.25) |
| DOAC | 106 | 5036 | 2.10 (1.72–2.53) | 15 | 395 | 3.80 (2.19–6.06) | 1.21 (0.98–1.48) | 1.08 (0.88–1.33) | 2.16 (1.29–3.60) | | 1.63 (0.97–2.71) |
| Dual antiplatelet | 86 | 2622 | 3.28 (2.63–4.02) | 7 | 141 | 4.95 (2.13–9.57) | 1.89 (1.51–2.37) | 1.36 (1.08–1.71) | 2.79 (1.32–5.88) | | 1.89 (0.90–3.98) |
| VKA/DOAC + antiplatelet | 317 | 6668 | 4.75 (4.25–5.30) | 39 | 401 | 9.71 (6.98–13.09) | 2.74 (2.39–3.14) | 2.33 (2.03–2.67) | 5.46 (3.95–7.55) | | 4.19 (3.02–5.80) |
| VKA/DOAC triple therapy | 21 | 255 | 8.24 (5.20–12.28) | 6 | 15 | 39.02 (15.51–79.05) | 4.70 (3.04–7.26) | 3.78 (2.45–5.85) | 21.26 (9.51–47.53) | | 14.49 (6.47–32.47) |
| **Skin cancer** | | | | | | | | | | | |
| All patients | 364 | 16397 | 2.22 (2.00–2.46) | 45 | 1650 | 2.73 (2.01–3.60) |  |  |  | |  |
| No antithrombotic therapy | 99 | 5863 | 1.69 (2.38–2.04) | 18 | 562 | 3.20 (1.94–4.92) | Reference | Reference | 1.89 (1.14–3.12) | | 1.69 (1.02–2.79) |
| Antithrombotic therapy | 265 | 10534 | 2.52 (2.22–2.83) | 27 | 1088 | 2.48 (1.66–3.54) | 1.49 (1.18–1.88) | 1.30 (1.03–1.64) | 1.47 (0.96–2.25) | | 1.13 (0.74–1.74) |
| Antiplatelet | 85 | 3619 | 2.35 (1.88–2.88) | 10 | 321 | 3.11 (1.56–5.46) | 1.39 (1.04–1.86) | 1.15 (0.85–1.54) | 1.84 (0.96–3.52) | | 1.33 (0.69–2.55) |
| VKA | 95 | 4547 | 2.09 (1.70–2.54) | 11 | 547 | 2.01 (1.04–3.44) | 1.24 (0.94–1.64) | 1.18 (0.89–1.57) | 1.19 (0.64–2.23) | | 0.99 (0.53–1.85) |
| DOAC | 14 | 825 | 1.70 (0.96–2.75) | # | # | 3.51 (0.87–9.11) | 1.00 (0.57–1.75) | 0.91 (0.52–1.59) | 2.08 (0.66–6.55) | | 1.93 (0.61–6.11) |
| Dual antiplatelet | 15 | 439 | 3.41 (1.96–5.44) | # | # | 2.88 (0.16–12.68) | 2.02 (1.17–3.48) | 1.52 (0.88–2.63) | 1.70 (0.24–12.16) | | 1.16 (0.16–8.31) |
| VKA/DOAC + antiplatelet | 53 | 1068 | 4.96 (3.74–6.42) | # | # | 2.11 (0.35–6.51) | 2.94 (2.11–4.11) | 2.45 (1.74–3.45) | 1.25 (0.31–5.05) | | 0.85 (0.21–3.44) |
| VKA/DOAC triple therapy | # | # | 8.66 (2.15–22.44) | # | # | NP | 5.09 (1.61–16.05) | 4.03 (1.27–12.79) | NP | | NP |
| **Intracranial cancers** |  |  |  |  |  |  |  |  |  | |  |
| All patients | 166 | 6740 | 2.46 (2.11–2.86) | 18 | 283 | 6.36 (3.85–9.76) |  |  |  | |  |
| No antithrombotic therapy | 40 | 2440 | 1.64 (1.18–2.20) | 10 | 127 | 7.85 (3.93–13.77) | Reference | Reference | 4.84 (2.52–9.72) | | 5.36 (2.66–10.80) |
| Antithrombotic therapy | 136 | 4299 | 2.93 (2.45–3.47) | 8 | 156 | 5.14 (2.35–9.56) | 1.79 (1.25–2.55) | 1.58 (1.10–2.26) | 3.12 (1.45–6.69) | | 2.95 (1.37–6.35) |
| Antiplatelet | 31 | 1458 | 2.13 (1.46–2.96) | # | # | 6.29 (1.56–16.30) | 1.29 (0.81–2.07) | 1.05 (0.65–1.69) | 3.84 (1.19–12.45) | | 3.65 (1.12–11.88) |
| VKA | 53 | 1833 | 2.89 (2.18–3.74) | # | # | 1.48 (0.08–6.51) | 1.77 (1.17–2.66) | 1.74 (1.15–2.62) | 0.91 (0.12–6.59) | | 0.87 (0.12–6.34) |
| DOAC | 6 | 339 | 1.77 (0.70–3.59) | # | # | 4.32 (0.25–18.99) | 1.08 (0.46–2.55) | 0.93 (0.39–2.21) | 2.62 (0.36–19.06) | | 3.00 (0.41–22.07) |
| Dual antiplatelet | 9 | 196 | 4.58 (2.20–8.26) | # | # | NP | 2.79 (1.36–5.76) | 2.13 (1.02–4.42) | NP | | NP |
| VKA/DOAC + antiplatelet | 26 | 464 | 5.61 (3.72–8.04) | # | # | 34.10 (8.48–88.40) | 3.43 (2.09–5.62) | 3.09 (1.86–5.12) | 19.55 (6.00–63.67) | | 19.71 (6.01–64.68) |
| VKA/DOAC triple therapy | # | # | 10.47 (0.60–46.05) | # | # | NP | 6.22 (0.85–45.31) | 5.92 (0.81–43.42) | NP | | NP |
| **Hematological cancers** | | | | | | | | | | | |
| All patients | 749 | 33633 | 2.23 (2.07–2.39) | 133 | 2626 | 5.06 (4.25–5.97) |  |  |  | |  |
| No antithrombotic therapy | 185 | 12129 | 1.53 (1.32–1.76) | 60 | 1079 | 5.56 (4.27–7.09) | Reference | Reference | 3.61 (2.70–4.83) | | 3.18 (2.37–4.26) |
| Antithrombotic therapy | 564 | 21505 | 2.62 (2.42–2.85) | 73 | 1548 | 4.72 (3.72–5.88) | 1.72 (1.46–2.03) | 1.53 (1.29–1.81) | 3.07 (2.34–4.02) | | 2.60 (1.98–3.41) |
| Antiplatelet | 173 | 7327 | 2.36 (2.03–2.73) | 24 | 498 | 4.82 (3.14–7.02) | 1.55 (1.26–1.90) | 1.25 (1.01–1.54) | 3.13 (2.05–4.79) | | 2.50 (1.63–3.83) |
| VKA | 217 | 9286 | 2.34 (2.04–2.66) | 26 | 695 | 3.74 (2.48–5.37) | 1.53 (1.26–1.87) | 1.54 (1.26–1.87) | 2.44 (1.62–3.67) | | 2.28 (1.51–3.44) |
| DOAC | 32 | 1618 | 1.98 (1.37–2.74) | 7 | 153 | 4.58 (1.97–8.86) | 1.29 (0.89–1.88) | 1.21 (0.83–1.76) | 2.99 (1.40–6.35) | | 2.45 (1.15–5.22) |
| Dual antiplatelet | 34 | 873 | 3.90 (2.73–5.36) | 5 | 50 | 10.10 (3.62–21.71) | 2.55 (1.77–3.68) | 1.87 (1.29–2.71) | 6.54 (2.69–15.90) | | 5.17 (2.13–12.59) |
| VKA/DOAC + antiplatelet | 104 | 2329 | 4.47 (3.66–5.38) | 10 | 147 | 6.81 (3.41–11.94) | 2.92 (2.30–3.71) | 2.57 (2.01–3.28) | 4.42 (2.34–8.35) | | 3.53 (1.86–6.68) |
| VKA/DOAC triple therapy | 4 | 72 | 5.55 (1.72–12.90) | # | # | 16.80 (0.96–73.90) | 3.61 (1.34–9.73) | 2.91 (1.08–7.85) | 10.47 (1.47–74.72) | | 8.13 (1.13–58.26) |
| **Other malignancies** | | | | | | | | | | | |
| All patients | 219 | 9171 | 2.39 (2.09–2.72) | 28 | 595 | 4.70 (3.17–6.67) |  |  |  | |  |
| No antithrombotic therapy | 72 | 3263 | 2.21 (1.74–2.76) | 11 | 243 | 4.52 (2.35–7.75) | Reference | Reference | 1.99 (1.06–3.76) | | 2.09 (1.10–3.95) |
| Antithrombotic therapy | 147 | 5909 | 2.49 (2.11–2.91) | 17 | 352 | 4.82 (2.88–7.49) | 1.12 (0.85–1.49) | 0.99 (0.75–1.32) | 2.13 (1.25–3.61) | | 1.93 (1.13–3.28) |
| Antiplatelet | 42 | 2053 | 2.05 (1.49–2.73) | 4 | 114 | 3.51 (1.09–8.15) | 0.93 (0.63–1.36) | 0.76 (0.51–1.11) | 1.55 (0.57–4.25) | | 1.29 (0.47–3.54) |
| VKA | 56 | 2461 | 2.28 (1.73–2.92) | 7 | 171 | 4.09 (1.76–7.91) | 1.03 (0.73–1.46) | 1.00 (0.70–1.42) | 1.83 (0.84–3.97) | | 1.87 (0.86–4.09) |
| DOAC | 16 | 482 | 3.32 (1.95–5.23) | # | # | 3.65 (0.21–16.04) | 1.49 (0.86–2.56) | 1.25 (0.73–2.16) | 1.60 (0.22–11.49) | | 1.32 (0.18–9.52) |
| Dual antiplatelet | 4 | 239 | 1.67 (0.52–3.89) | # | # | 10.80 (0.62–47.52) | 0.75 (0.27–2.05) | 0.57 (0.21–1.58) | 4.55 (0.63–32.73) | | 3.58 (0.50–25.84) |
| VKA/DOAC + antiplatelet | 24 | 655 | 3.66 (2.39–5.33) | 4 | 28 | 14.41 (4.47–33.48) | 1.65 (1.04–2.63) | 1.49 (0.93–2.39) | 6.12 (2.23–16.77) | | 5.45 (0.50–25.84) |
| VKA/DOAC triple therapy | 5 | 19 | 26.31 (9.43–56.55) | # | # | NP | 11.53 (4.65–28.55) | 10.46 (4.20–26.06) | NP | | NP |

* adjusted for age, sex, ischemic heart disease, valvular heart disease, diabetes, hypertension, liver disease and kidney failure

# because of Danish privacy regulations, censoring occurred when the number of persons or events was less than four

Abbreviations: AF (atrial fibrillation), AFL (atrial flutter), CI (confidence interval), DOAC (direct oral anticoagulant), HR (hazard ratio), MB (major bleeding), NP (not possible), pyrs (person-years), VKA (vitamin K antagonists)

**Supplementary Table S6** Sites of bleeding according to cancer type and antithrombotic therapy in patients with AF/AFL with active cancer

|  | **Active cancer** | | | | | | |
| --- | --- | --- | --- | --- | --- | --- | --- |
|  | MB | GI | Cerebral | Urinary | Respiratory | No class |  |
| **All cancer** | | | | | | | |
| All patients | 1447 | 502 | 190 | 469 | 202 | 110 |  |
| No antithrombotic therapy | 500 | 170 | 76 | 164 | 68 | 27 |  |
| Antithrombotic therapy | 947 | 332 | 114 | 305 | 134 | 83 |  |
| Antiplatelet | 265 | 108 | 19 | 77 | 40 | 27 |  |
| VKA | 383 | 123 | 56 | 129 | 54 | 24 |  |
| DOAC | 71 | 19 | 10 | 29 | 7 | 7 |  |
| Dual antiplatelet | 45 | 19 | 7 | 9 | 4 | 7 |  |
| VKA + antiplatelet | 159 | 48 | 21 | 55 | 26 | 15 |  |
| DOAC + antiplatelet | 13 | 8 | 1 | 5 | 0 | 2 |  |
| VKA/DOAC triple therapy | 11 | 7 | 0 | 1 | 3 | 1 |  |
| **Respiratory and intrathoracic cancers** | | | | | | | |
| All patients | 239 | 86 | 20 | 29 | 94 | 12 |  |
| No antithrombotic therapy | 83 | 26 | 7 | 9 | 37 | 6 |  |
| Antithrombotic therapy | 156 | 60 | 13 | 20 | 57 | 6 |  |
| **Breast cancer** | | | | | | | |
| All patients | 87 | 35 | 23 | 10 | 13 | 7 |  |
| No antithrombotic therapy | 24 | 12 | 6 | 3 | 1 | 2 |  |
| Antithrombotic therapy | 63 | 23 | 17 | 7 | 12 | 5 |  |
| **Urogenital cancers** | | | | | | | |
| All patients | 624 | 144 | 55 | 364 | 34 | 36 |  |
| No antithrombotic therapy | 205 | 39 | 22 | 131 | 10 | 4 |  |
| Antithrombotic therapy | 419 | 105 | 33 | 233 | 24 | 32 |  |
| **Gastrointestinal cancers** | | | | | | | |
| All patients | 403 | 218 | 53 | 56 | 43 | 44 |  |
| No antithrombotic therapy | 150 | 87 | 20 | 18 | 13 | 14 |  |
| Antithrombotic therapy | 253 | 131 | 33 | 38 | 30 | 30 |  |
| **Skin cancer** | | | | | | | |
| All patients | 45 | 12 | 13 | 13 | 7 | 1 |  |
| No antithrombotic therapy | 18 | 6 | 7 | 5 | 0 | 0 |  |
| Antithrombotic therapy | 27 | 6 | 6 | 8 | 7 | 1 |  |
| **Intracranial cancers** |  |  |  |  |  |  |  |
| All patients | 18 | 5 | 5 | 4 | 2 | 2 |  |
| No antithrombotic therapy | 10 | 2 | 2 | 4 | 1 | 1 |  |
| Antithrombotic therapy | 8 | 3 | 3 | 0 | 1 | 1 |  |
| **Hematological cancers** | | | | | | | |
| All patients | 133 | 43 | 34 | 29 | 19 | 10 |  |
| No antithrombotic therapy | 60 | 20 | 15 | 11 | 12 | 3 |  |
| Antithrombotic therapy | 73 | 23 | 19 | 18 | 7 | 7 |  |
| **Other malignancies** | | | | | | | |
| All patients | 28 | 11 | 8 | 3 | 2 | 5 |  |
| No antithrombotic therapy | 11 | 3 | 4 | 1 | 1 | 2 |  |
| Antithrombotic therapy | 17 | 8 | 4 | 2 | 1 | 3 |  |

Abbreviations: AF (atrial fibrillation), AFL (atrial flutter), DOAC (direct oral anticoagulant), GI (gastrointestinal), MB (major bleeding), VKA (vitamin K antagonists)

**Supplementary Table S7** Hazard ratios of thromboembolism associated with antithrombotic therapy in patients with AF/AFL **with** **active cancer.** Matched patients with AF/AFL without cancer serve as a reference.

|  | **No cancer** | | | **Active cancer** | | | **No cancer** | | **Active cancer** | | |
| --- | --- | --- | --- | --- | --- | --- | --- | --- | --- | --- | --- |
|  | TE | Person  years | Incidence rate per 100 pyrs  (95% CI) | TE | Person  years | Incidence rate per 100 pyrs  (95% CI) | Crude hazard ratio  (95% CI) | Adjusted hazard ratio  (95% CI)* | Crude hazard ratio  (95% CI) | Adjusted hazard ratio  (95% CI)* |  |
| **All cancer** | | | | | | | | | | | |
| All patients | 11457 | 422976 | 2.71 (2.66–2.76) | 1067 | 27271 | 3.91 (3.68–4.15) |  |  |  |  |  |
| No antithrombotic therapy | 4033 | 153675 | 2.62 (2.54–2.71) | 435 | 10374 | 4.19 (3.81–4.60) | Reference | Reference | 1.56 (1.42–1.73) | 1.45 (1.31–1.60) |  |
| Antithrombotic therapy | 7424 | 269301 | 2.76 (2.69–2.82) | 632 | 16897 | 3.74 (3.46–4.04) | 1.05 (1.01–1.09) | 0.91 (0.87–0.94) | 1.40 (1.29–1.52) | 1.17 (1.08–1.27) |  |
| Antiplatelet | 3532 | 92319 | 3.83 (3.70–3.95) | 266 | 5465 | 4.87 (4.31–5.48) | 1.45 (1.39–1.52) | 1.14 (1.09–1.20) | 1.82 (1.61–2.06) | 1.40 (1.24–1.59) |  |
| VKA | 1994 | 116137 | 1.72 (1.64–1.79) | 211 | 7557 | 2.79 (2.43–3.19) | 0.66 (0.62–0.69) | 0.64 (0.61–0.68) | 1.05 (0.91–1.20) | 0.96 (0.84–1.11) |  |
| DOAC | 418 | 21106 | 1.98 (1.80–2.18) | 49 | 1486 | 3.30 (2.46–4.31) | 0.74 (0.67–0.82) | 0.68 (0.62–0.75) | 1.23 (0.93–1.63) | 1.09 (0.82–1.45) |  |
| Dual antiplatelet | 676 | 10281 | 6.58 (6.09–7.08) | 41 | 559 | 7.33 (5.32–9.81) | 2.48 (2.29–2.69) | 1.77 (1.63–1.91) | 2.71 (1.99–3.69) | 1.97 (1.45–2.68) |  |
| VKA + antiplatelet | 711 | 26793 | 2.65 (2.46–2.85) | 60 | 1652 | 3.63 (2.79–4.63) | 1.00 (0.93–1.09) | 0.83 (0.77–0.90) | 1.35 (1.05–1.75) | 1.05 (0.81–1.36) |  |
| DOAC + antiplatelet | 49 | 1818 | 2.69 (2.01–3.52) | 4 | 116 | 3.45 (1.07–8.00) | 0.99 (0.75–1.31) | 0.75 (0.57–1.00) | 1.27 (0.48–3.38) | 0.93 (0.35–2.47) |  |
| VKA/DOAC triple therapy | 44 | 847 | 5.20 (3.81–6.89) | # | # | 1.63 (0.09–7.16) | 1.98 (1.48–2.64) | 1.57 (1.18–2.10) | 0.59 (0.08–4.18) | 0.43 (0.06–3.08) |  |
| **Respiratory and intrathoracic cancers** | | | | | | | | | | |  |
| All patients | 2020 | 72054 | 2.80 (2.68–2.93) | 193 | 4063 | 4.75 (4.11–5.45) |  |  |  |  |  |
| No antithrombotic therapy | 710 | 26360 | 2.69 (2.50–2.90) | 72 | 1641 | 4.39 (3.45–5.48) | Reference | Reference | 1.56 (1.22–2.01) | 1.53 (1.19–1.97) |  |
| Antithrombotic therapy | 1310 | 45694 | 2.87 (2.71–3.02) | 121 | 2290 | 5.28 (4.40–6.28) | 1.06 (0.97–1.16) | 0.93 (0.85–1.02) | 1.88 (1.55–2.28) | 1.69 (1.39–2.05) |  |
| Antiplatelet | 637 | 15843 | 4.02 (3.72–4.34) | 54 | 803 | 6.72 (5.09–8.68) | 1.59 (1.34–1.66) | 1.19 (1.07–1.33) | 2.39 (1.81–3.15) | 2.00 (1.52–2.64) |  |
| VKA | 330 | 19319 | 1.71 (1.53–1.90) | 34 | 921 | 3.69 (2.59–5.08) | 0.63 (0.56–0.72) | 0.62 (0.55–0.71) | 1.32 (0.94–1.85) | 1.29 (0.91–1.82) |  |
| DOAC | 87 | 3753 | 2.32 (1.86–2.84) | 11 | 242 | 4.54 (2.36–7.78) | 0.85 (0.68–1.06) | 0.79 (0.63–0.98) | 1.63 (0.90–2.96) | 1.44 (0.80–2.62) |  |
| Dual antiplatelet | 123 | 1723 | 7.14 (5.95–8.48) | 8 | 84 | 9.49 (4.34–17.67) | 2.63 (2.17–3.18) | 1.91 (1.57–2.31) | 3.29 (1.64–6.61) | 2.76 (1.37–5.54) |  |
| VKA/DOAC + antiplatelet | 126 | 4914 | 2.56 (2.14–3.04) | 14 | 230 | 6.08 (3.42–9.84) | 0.95 (0.79–1.15) | 0.78 (0.64–0.94) | 2.15 (1.27–3.65) | 1.81 (1.07–3.08) |  |
| VKA/DOAC triple therapy | 7 | 143 | 4.90 (2.11–9.48) | # | # | 0 | 1.76 (0.84–3.71) | 1.35 (0.64–2.84) | NP | NP |  |
| **Breast cancer** | | | | | | | | | | |  |
| All patients | 946 | 33186 | 2.85 (2.67–3.04) | 108 | 3488 | 3.10 (2.55–3.71) |  |  |  |  |  |
| No antithrombotic therapy | 374 | 12132 | 3.08 (2.78–3.41) | 43 | 1354 | 3.18 (2.32–4.22) | Reference | Reference | 1.03 (0.75–1.41) | 0.98 (0.71–1.35) |  |
| Antithrombotic therapy | 572 | 21054 | 2.72 (2.50–2.95) | 65 | 1234 | 3.05 (2.36–3.85) | 0.88 (0.77–1.00) | 0.76 (0.67–0.87) | 0.99 (0.76–1.28) | 0.78 (0.59–1.02) |  |
| Antiplatelet | 271 | 7266 | 3.73 (3.30–4.19) | 27 | 720 | 3.75 (2.51–5.35) | 1.21 (1.03–1.41) | 0.93 (0.80–1.10) | 1.21 (0.82–1.79) | 0.88 (0.60–1.32) |  |
| VKA | 157 | 9063 | 1.73 (1.48–2.02) | 22 | 952 | 2.31 (1.47–3.41) | 0.56 (0.47–0.68) | 0.56 (0.46–0.67) | 0.75 (0.49–1.15) | 0.66 (0.43–1.03) |  |
| DOAC | 32 | 1598 | 2.00 (1.39–2.78) | 8 | 192 | 4.16 (1.90–7.74) | 0.65 (0.45–0.93) | 0.60 (0.42–0.86) | 1.35 (0.67–2.71) | 1.21 (0.60–2.44) |  |
| Dual antiplatelet | 51 | 806 | 6.33 (4.74–8.22) | # | # | 4.24 (1.05–11.00) | 2.05 (1.52–2.74) | 1.45 (1.08–1.95) | 1.38 (0.44–4.30) | 0.91 (0.29–2.84) |  |
| VKA/DOAC + antiplatelet | 59 | 2244 | 2.62 (2.01–3.36) | 5 | 196 | 2.56 (0.92–5.50) | 0.85 (0.65–1.12) | 0.69 (0.52–0.91) | 0.83 (0.34–2.00) | 0.62 (0.26–1.50) |  |
| VKA/DOAC triple therapy | # | # | 2.61 (0.43–8.05) | # | # | NP | 0.84 (0.21–3.35) | 0.71 (0.18–2.86) | NP | NP |  |
| **Urogenital cancers** | | | | | | | | | | |  |
| All patients | 2970 | 110237 | 2.69 (2.60–2.79) | 335 | 10011 | 3.35 (3.00–3.72) |  |  |  |  |  |
| No antithrombotic therapy | 1041 | 30093 | 2.60 (2.44–2.76) | 136 | 3526 | 3.86 (3.24–4.54) | Reference | Reference | 1.47 (1.23–1.76) | 1.35 (1.13–1.62) |  |
| Antithrombotic therapy | 1929 | 70143 | 2.75 (2.63–2.87) | 199 | 6485 | 3.07 (2.66–3.62) | 1.06 (0.98–1.14) | 0.91 (0.85–0.99) | 1.17 (1.01–1.37) | 0.99 (0.85–1.16) |  |
| Antiplatelet | 919 | 24203 | 3.80 (3.56–4.05) | 86 | 1998 | 4.30 (3.46–5.28) | 1.46 (1.34–1.60) | 1.15 (1.05–1.26) | 1.65 (1.32–2.05) | 0.69 (0.43–0.90) |  |
| VKA | 518 | 30105 | 1.72 (2.58–1.87) | 58 | 2972 | 1.95 (1.49–2.50) | 0.66 (0.60–0.74) | 0.64 (0.58–0.72) | 0.75 (0.57–0.98) | 1.28 (1.02–1.59) |  |
| DOAC | 105 | 5470 | 1.92 (1.58–2.31) | 13 | 499 | 2.61 (1.43–4.29) | 0.74 (0.60–0.90) | 0.67 (0.55–0.83) | 0.99 (0.57–1.72) | 0.91 (0.53–0.90) |  |
| Dual antiplatelet | 168 | 2643 | 6.36 (5.44–7.37) | 18 | 218 | 8.24 (5.00–12.65) | 2.44 (2.07–2.87) | 1.76 (1.49–2.07) | 3.13 (1.97–4.99) | 2.27 (1.42–3.62) |  |
| VKA/DOAC + antiplatelet | 206 | 7500 | 2.75 (2.39–3.14) | 24 | 770 | 3.12 (2.03–4.54) | 1.06 (0.91–1.23) | 0.86 (0.75–1.01) | 1.19 (0.80–1.79) | 0.92 (0.61–1.38) |  |
| VKA/DOAC triple therapy | 13 | 223 | 5.83 | # | # | NP | 2.20 (1.27–3.80) | 1.80 (1.04–3.11) | NP | NP |  |
| **Gastrointestinal cancers** | | | | | | | | | | |  |
| All patients | 2629 | 98738 | 2.66 (2.56–2.77) | 326 | 6770 | 4.82 (4.31–5.36) |  |  |  |  |  |
| No antithrombotic therapy | 890 | 35896 | 2.48 (2.32–2.65) | 149 | 2729 | 5.46 (4.63–6.38) | Reference | Reference | 2.13 (1.79–2.54) | 1.82 (1.53–2.17) |  |
| Antithrombotic therapy | 1739 | 62841 | 2.77 (2.64–2.90) | 177 | 4041 | 4.38 (3.77–5.06) | 1.11 (1.03–1.21) | 0.96 (0.89–1.04) | 1.72 (1.47–2.02) | 1.35 (1.15–1.59) |  |
| Antiplatelet | 800 | 21395 | 3.74 (3.49–4.00) | 65 | 1335 | 4.87 (3.78–6.15) | 1.51 (1.37–1.66) | 1.19 (1.08–1.31) | 1.91 (1.49–2.46) | 1.40 (1.09–1.80) |  |
| VKA | 485 | 27228 | 1.78 (1.63–1.94) | 66 | 1781 | 3.71 (2.88–4.67) | 0.72 (0.64–0.80) | 0.70 (0.63–0.78) | 1.46 (1.14–1.87) | 1.26 (0.98–1.62) |  |
| DOAC | 108 | 4989 | 2.16 (1.78–2.60) | 18 | 378 | 4.77 (2.89–7.32) | 0.86 (0.71–1.05) | 0.78 (0.64–0.95) | 1.87 (1.17–2.99) | 1.54 (0.97–2.46) |  |
| Dual antiplatelet | 165 | 2438 | 6.77 (5.79–7.85) | 9 | 131 | 6.88 (3.31–12.41) | 2.70 (2.29–3.92) | 1.88 (1.59–2.23) | 2.67 (1.38–5.14) | 1.79 (0.93–3.45) |  |
| VKA/DOAC + antiplatelet | 169 | 6580 | 2.57 (2.20–2.98) | 19 | 403 | 4.72 (2.90–7.16) | 1.03 (0.88–1.22) | 0.85 (0.72–1.00) | 1.84 (1.17–2.89) | 1.39 (0.88–2.19) |  |
| VKA/DOAC triple therapy | 12 | 212 | 5.67 (3.03–9.51) | # | # | NP | 2.22 (1.25–3.92) | 1.72 (0.97–3.04) | NP | NP |  |
| **Skin cancer** | | | | | | | | | | |  |
| All patients | 422 | 16344 | 2.58 (2.34–2.84) | 39 | 1646 | 3.27 (1.70–3.19) |  |  |  |  |  |
| No antithrombotic therapy | 150 | 5891 | 2.55 (2.16–2.97) | 8 | 574 | 1.39 (0.64–2.60) | Reference | Reference | 0.55 (0.27–1.11) | 0.51 (0.25–1.04) |  |
| Antithrombotic therapy | 272 | 10453 | 2.60 (2.30–2.92) | 31 | 1073 | 2.89 (1.99–4.03) | 1.02 (0.84–1.25) | 0.87 (0.71–1.07) | 1.13 (0.77–1.66) | 0.92 (0.62–1.36) |  |
| Antiplatelet | 114 | 3601 | 3.17 (2.62–3.78) | 17 | 319 | 5.33 (3.18–8.29) | 1.24 (0.97–1.59) | 0.96 (0.75–1.23) | 2.08 (1.26–3.44) | 1.55 (0.93–2.56) |  |
| VKA | 76 | 4551 | 1.67 (1.32–2.07) | 9 | 555 | 1.65 (0.79–2.98) | 0.66 (0.50–0.86) | 0.64 (0.49–0.85) | 0.65 (0.33–1.27) | 0.58 (0.30–1.15) |  |
| DOAC | 15 | 813 | 1.84 (1.06–2.94) | # | # | NP | 0.71 (0.42–1.22) | 0.66 (0.39–1.12) | NP | NP |  |
| Dual antiplatelet | 36 | 403 | 8.93 (6.32–12.17) | # | # | 6.38 (1.06–19.69) | 3.46 (2.40–4.97) | 2.47 (1.71–3.57) | 2.46 (0.61–9.94) | 1.61 (0.40–6.50) |  |
| VKA/DOAC + antiplatelet | 30 | 1054 | 2.85 (1.95–3.99) | # | # | 3.30 (0.82–8.56) | 1.12 (0.75–1.65) | 0.89 (0.60–1.33) | 1.29 (0.41–4.05) | 0.88 (0.28–2.76) |  |
| VKA/DOAC triple therapy | # | # | 3.29 (0.19–14.47) | # | # | NP | 1.30 (0.18–9.27) | 0.91 (0.13–6.50) | NP | NP |  |
| **Intracranial cancers** | | | | | | | | | | |  |
| All patients | 195 | 6726 | 2.90 (2.51–3.33) | 12 | 291 | 4.12 (2.21–6.91) |  |  |  |  |  |
| No antithrombotic therapy | 68 | 2451 | 2.77 (2.17–3.49) | 5 | 132 | 3.80 (1.36–8.16) | Reference | Reference | 1.35 (0.54–3.34) | 1.54 (0.62–3.83) |  |
| Antithrombotic therapy | 127 | 4275 | 2.97 (2.48–3.52) | 7 | 160 | 4.38 (1.88–8.47) | 1.07 (0.80–1.44) | 0.92 (0.68–1.24) | 1.54 (0.71–3.37) | 1.54 (0.70–3.38) |  |
| Antiplatelet | 75 | 1444 | 5.19 (4.11–6.46) | # | # | 5.80 (1.44–15.03) | 1.87 (1.35–2.59) | 1.42 (1.01–1.98) | 2.07 (0.65–6.60) | 1.99 (0.62–6.35) |  |
| VKA | 31 | 1844 | 1.68 (1.16–2.34) | # | # | 2.98 (0.50–9.19) | 0.61 (0.40–0.93) | 0.61 (0.40–0.93) | 1.05 (0.26–4.30) | 1.04 (0.25–4.26) |  |
| DOAC | 4 | 335 | 1.19 (0.37–2.77) | # | # | 8.53 (1.42–26.32) | 0.43 (0.16–1.17) | 0.37 (0.13–1.01) | 2.87 (0.73–12.15) | 3.38 (0.82–14.05) |  |
| Dual antiplatelet | 4 | 185 | 2.16 (0.67–5.02) | # | # | NP | 0.78 (0.28–2.13) | 0.55 (0.20–1.52) | NP | NP |  |
| VKA/DOAC + antiplatelet | 13 | 459 | 2.84 (1.56–4.67) | # | # | NP | 1.02 (0.56–1.85) | 0.86 (0.47–1.56) | NP | NP |  |
| VKA/DOAC triple therapy | # | # | NP | # | # | NP | NP | NP | NP | NP |  |
| **Hematological cancers** | | | | | | | | | | |  |
| All patients | 890 | 33542 | 2.65 (2.48–2.83) | 129 | 2620 | 4.92 (4.12–5.82) |  |  |  |  |  |
| No antithrombotic therapy | 294 | 12186 | 2.41 (2.15–2.70) | 58 | 1097 | 5.29 (4.04–6.77) | Reference | Reference | 2.14 (1.62–2.84) | 2.01 (1.52–2.67) |  |
| Antithrombotic therapy | 596 | 21356 | 2.79 (2.57–3.02) | 71 | 1523 | 4.66 (3.66–5.83) | 1.15 (1.00–1.33) | 1.00 (0.87–1.15) | 1.91 (1.47–2.47) | 1.65 (1.27–2.14) |  |
| Antiplatelet | 293 | 7312 | 4.01 (3.57–4.48) | 30 | 492 | 6.10 (4.17–8.55) | 1.66 (1.41–1.95) | 1.27 (1.08–1.50) | 2.49 (1.71–3.62) | 1.95 (1.34–2.84) |  |
| VKA | 164 | 9287 | 1.77 (1.51–2.05) | 31 | 687 | 4.52 (3.11–6.30) | 0.73 (0.60–0.89) | 0.73 (0.61–0.89) | 1.86 (1.28–2.69) | 1.79 (1.23–2.59) |  |
| DOAC | 25 | 1600 | 1.56 (1.03–2.26) | # | # | 1.94 (0.48–5.04) | 0.64 (0.43–0.96) | 0.61 (0.40–0.91) | 0.80 (0.26–2.49) | 0.70 (0.23–2.19) |  |
| Dual antiplatelet | 45 | 810 | 5.55 (4.09–7.34) | # | # | 7.22 (1.79–18.70) | 2.29 (1.67–3.13) | 1.58 (1.15–2.18) | 2.93 (0.94–9.13) | 2.40 (2.77–2.48) |  |
| VKA/DOAC + antiplatelet | 67 | 2288 | 2.93 (2.28–3.69) | # | # | 2.09 (0.52–5.42) | 1.21 (0.93–1.57) | 0.98 (0.75–1.28) | 0.85 (0.27–2.65) | 0.65 (0.21–2.02) |  |
| VKA/DOAC triple therapy | # | # | 3.42 (0.57–10.55) | # | # | 18.10 (1.03–79.62) | 1.37 (0.34–5.52) | 1.04 (0.26–4.18) | 7.07 (0.99–50.38) | 4.49 (0.63–32.07) |  |
| **Other malignancies** | | | | | | | | | | |  |
| All patients | 256 | 9147 | 2.80 (2.47–3.16) | 20 | 606 | 3.30 (2.06–4.97) |  |  |  |  |  |
| No antithrombotic therapy | 90 | 3286 | 2.74 (2.21–3.34) | 8 | 249 | 3.22 (1.47–6.00) | Reference | Reference | 1.17 (0.57–2.40) | 1.19 (0.57–2.45) |  |
| Antithrombotic therapy | 166 | 5861 | 2.83 (2.42–3.29) | 12 | 357 | 3.36 (1.80–5.63) | 1.03 (0.80–1.34) | 0.89 (0.69–1.15) | 1.20 (0.66–2.19) | 1.05 (0.57–1.92) |  |
| Antiplatelet | 72 | 2039 | 3.53 (2.78–4.41) | 8 | 117 | 6.83 (3.12–12.71) | 1.29 (0.95–1.76) | 1.04 (0.76–1.43) | 2.42 (1.17–4.99) | 1.85 (0.89–3.84) |  |
| VKA | 42 | 2464 | 1.70 (1.24–2.27) | # | # | 1.71 (0.42–4.43) | 0.62 (0.43–0.90) | 0.59 (0.41–0.86) | 0.62 (0.20–1.95) | 0.71 (0.19–1.92) |  |
| DOAC | 6 | 470 | 1.28 (0.51–2.59) | # | # | 0 | 0.46 90.2–1.06) | 0.40 (0.18–0393) | NP | NP |  |
| Dual antiplatelet | 21 | 221 | 9.50 (5.99–14.16) | # | # | 11.97 (0.68–52.66) | 3.47 (2.15–5.57) | 2.58 (1.60–4.17) | 4.27 (0.59–30.65) | 3.68 (0.51–26.46) |  |
| VKA/DOAC + antiplatelet | 23 | 650 | 3.54 (2.28–5.19) | # | # | NP | 1.29 (0.82–2.04) | 1.07 (0.67–1.70) | NP | NP |  |
| VKA/DOAC triple therapy | # | # | 11.5 (1.89–35.01) | # | # | NP | 3.95 (0.97–16.06) | 3.68 (0.90–14.99) | NP | NP |  |

* adjusted for age, sex, ischemic heart disease, valvular heart disease, diabetes, hypertension, liver disease and kidney failure

# because of Danish privacy regulations, censoring occurred when the number of persons or events was less than four

Abbreviations: AF (atrial fibrillation), AFL (atrial flutter), CI (confidence interval), DOAC (direct oral anticoagulant), HR (hazard ratio), NP (not possible), pyrs (person-years), TE (thromboembolism), VKA (vitamin K antagonists)
